# Supplementary material for: The applicability of commonly used predictive scoring systems in Indigenous Australians with sepsis: An observational study
Source: PLoS One. 2020 Jul 22;15(7):e0236339. doi: 10.1371/journal.pone.0236339 (PMC7375531; doi:10.1371/journal.pone.0236339)
Supplement: Attachment — Submitted filename: PONE-D-20-14432_reviewer_comments.pdf [file pone.0236339.s002.pdf]

PLOS ONE

The applicability of commonly used predictive scoring systems in Indigenous Australians with sepsis: an observational study

--Manuscript Draft--

**Manuscript Number:**	PONE-D-20-14432
**Article Type:**	Research Article
**Full Title:**	The applicability of commonly used predictive scoring systems in Indigenous Australians with sepsis: an observational study
**Short Title:**	The utility of predictive scores in Indigenous Australians with sepsis
**Corresponding Author:**	Josh Hanson<br>The Kirby Institute<br>Sydney, NSW AUSTRALIA
**Keywords:**	Intensive Care Medicine; Indigenous Health; Sepsis; Tropical Medicine; Infectious Diseases; Prediction scores
**Abstract:**	Background: Indigenous Australians suffer a disproportionate burden of sepsis, however, the performance of scoring systems that predict mortality in Indigenous patients with critical illness is incompletely defined.<br>Materials and methods: The study was performed at an Australian tertiary-referral hospital between January 2014 and June 2017, and enrolled consecutive Indigenous and non-Indigenous adults admitted to ICU with sepsis. The ability of the ANZROD, APACHE-II, APACHE-III, SAPS-II, SOFA and qSOFA scores to predict death before ICU discharge in the two populations was compared.<br>Results : There were 442 individuals enrolled in the study, 145 (33%) identified as Indigenous. Indigenous patients were younger than non-Indigenous patients (median (interquartile range (IQR) 53 (43-60) versus 65 (52-73) years, p=0.0001) and comorbidity was more common (118/145 (81%) versus 204/297 (69%), p=0.005). Comorbidities that were more common in the Indigenous patients included diabetes mellitus (84/145 (58%) versus 67/297 (23%), p<0.0001), renal disease (56/145 (39%) versus 29/297 (10%), p<0.0001) and cardiovascular disease (58/145 (40%) versus 83/297 (28%), p=0.01). The use of supportive care (including vasopressors, mechanical ventilation and renal replacement therapy) was similar in Indigenous and non-Indigenous patients, and the two populations had an overall case-fatality rate that was comparable (17/145 (12%) and 38/297 (13%) (p=0.75)), although Indigenous patients died at a younger age (median (IQR): 54 (50-60) versus 70 (61-76) years, p=0.0001). There was no significant difference in the ability of any the scores to predict mortality in the two populations.<br>Conclusions : Although the crude case-fatality rates of Indigenous and non-Indigenous Australians admitted to ICU with sepsis is comparable, Indigenous patients die at a much younger age. Despite this, the ability of commonly used scoring systems to predict outcome in Indigenous Australians is similar to that of non-Indigenous Australians, supporting their use in ICUs with a significant Indigenous patient population and in clinical trials that enrol Indigenous Australians.
**Order of Authors:**	Josh Hanson
	Simon Smith
	James Brooks
	Taissa Groch
	Sayonne Sivalingam
	Venessa Curnow
	Angus Carter
	Satyen Hargovan
**Additional Information:**	
**Question**	**Response**

Powered by Editorial Manager® and ProduXion Manager® from Aries Systems Corporation

**Financial Disclosure**<br><br>Enter a financial disclosure statement that describes the sources of funding for the work included in this submission. Review the submission guidelines for detailed requirements. View published research articles from *PLOS ONE* for specific examples.<br><br>This statement is required for submission and **will appear in the published article** if the submission is accepted. Please make sure it is accurate.<br><br>**Unfunded studies**<br>Enter: *The author(s) received no specific funding for this work.*<br><br>**Funded studies**<br>Enter a statement with the following details:<br>• Initials of the authors who received each award<br>• Grant numbers awarded to each author<br>• The full name of each funder<br>• URL of each funder website<br>• Did the sponsors or funders play any role in the study design, data collection and analysis, decision to publish, or preparation of the manuscript?<br>• **NO** - Include this sentence at the end of your statement: *The funders had no role in study design, data collection and analysis, decision to publish, or preparation of the manuscript.*<br>• **YES** - Specify the role(s) played.<br><br>* typeset	The authors received no specific funding for this work.
**Competing Interests**<br><br>Use the instructions below to enter a competing interest statement for this submission. On behalf of all authors, disclose any competing interests that could be perceived to bias this work—acknowledging all financial support and any other relevant financial or non-financial competing interests.<br><br>This statement **will appear in the published article** if the submission is accepted. Please make sure it is	The authors have declared that no competing interests exist.

accurate. View published research articles from *PLOS ONE* for specific examples.<br><br>**NO authors have competing interests**<br><br>Enter: *The authors have declared that no competing interests exist.*<br><br>**Authors with competing interests**<br><br>Enter competing interest details beginning with this statement:<br><br>*I have read the journal's policy and the authors of this manuscript have the following competing interests: [insert competing interests here]*<br><br>* typeset	
**Ethics Statement**<br><br>Enter an ethics statement for this submission. This statement is required if the study involved:<br><br>• Human participants<br>• Human specimens or tissue<br>• Vertebrate animals or cephalopods<br>• Vertebrate embryos or tissues<br>• Field research<br><br>Write "N/A" if the submission does not require an ethics statement.<br><br>General guidance is provided below. Consult the submission guidelines for detailed instructions. **Make sure that all information entered here is included in the Methods section of the manuscript.**	Ethics approval was obtained from the Far North Queensland Human Research Ethics Committee (HREC/17/QCH/93/AMO2). As the data were retrospective and de-identified, the Committee waived the requirement for informed consent.

Powered by Editorial Manager® and ProduXion Manager® from Aries Systems Corporation

**Format for specific study types**<br><br>**Human Subject Research (involving human participants and/or tissue)**<br>• Give the name of the institutional review board or ethics committee that approved the study<br>• Include the approval number and/or a statement indicating approval of this research<br>• Indicate the form of consent obtained (written/oral) or the reason that consent was not obtained (e.g. the data were analyzed anonymously)<br><br>**Animal Research (involving vertebrate animals, embryos or tissues)**<br>• Provide the name of the Institutional Animal Care and Use Committee (IACUC) or other relevant ethics board that reviewed the study protocol, and indicate whether they approved this research or granted a formal waiver of ethical approval<br>• Include an approval number if one was obtained<br>• If the study involved *non-human primates*, add *additional details* about animal welfare and steps taken to ameliorate suffering<br>• If anesthesia, euthanasia, or any kind of animal sacrifice is part of the study, include briefly which substances and/or methods were applied<br><br>**Field Research**<br><br>Include the following details if this study involves the collection of plant, animal, or other materials from a natural setting:<br>• Field permit number<br>• Name of the institution or relevant body that granted permission	
**Data Availability**<br><br>Authors are required to make all data underlying the findings described fully available, without restriction, and from the time of publication. PLOS allows rare exceptions to address legal and ethical concerns. See the PLOS Data Policy and FAQ for detailed information.	Yes - all data are fully available without restriction

Powered by Editorial Manager® and ProduXion Manager® from Aries Systems Corporation

A Data Availability Statement describing where the data can be found is required at submission. Your answers to this question constitute the Data Availability Statement and **will be published in the article**, if accepted.<br><br>**Important:** Stating 'data available on request from the author' is not sufficient. If your data are only available upon request, select 'No' for the first question and explain your exceptional situation in the text box.<br><br>Do the authors confirm that all data underlying the findings described in their manuscript are fully available without restriction?	
**Describe where the data may be found in full sentences. If you are copying our sample text, replace any instances of XXX with the appropriate details.**<br><br>• If the data are **held or will be held in a public repository**, include URLs, accession numbers or DOIs. If this information will only be available after acceptance, indicate this by ticking the box below. For example: *All XXX files are available from the XXX database (accession number(s) XXX, XXX.*).<br>• If the data are all contained **within the manuscript and/or Supporting Information files**, enter the following: *All relevant data are within the manuscript and its Supporting Information files.*<br>• If neither of these applies but you are able to provide **details of access elsewhere**, with or without limitations, please do so. For example:<br><br>*Data cannot be shared publicly because of [XXX]. Data are available from the XXX Institutional Data Access / Ethics Committee (contact via XXX) for researchers who meet the criteria for access to confidential data.*<br><br>*The data underlying the results presented in the study are available from (include the name of the third party*	All relevant data are within the manuscript and its Supporting Information files. The final dataset has been submitted as an Excel file in this submission: "Final dataset for publication".

and contact information or URL).<br>• This text is appropriate if the data are owned by a third party and authors do not have permission to share the data.<br><br>* typeset	
Additional data availability information:	

Powered by Editorial Manager® and ProduXion Manager® from Aries Systems Corporation

Manuscript
Click here to download Manuscript Severity scores final PLoS One.docx

The applicability of commonly used predictive scoring systems in Indigenous Australians with sepsis: an observational study

Josh Hanson[1,2*], Simon Smith[1], James Brooks[3,4], Taissa Groch[5], Sayonne Sivalingam[3], Venessa Curnow[6], Angus Carter[3], Satyen Hargovan[3]

[1] Department of Medicine, Cairns Hospital, Cairns, Queensland, Australia

[2] The Kirby Institute, University of New South Wales, Sydney, New South Wales, Australia

[3] Department of Intensive Care Medicine, Cairns Hospital, Cairns, Queensland, Australia

[4] Department of Anaesthetics, Gloucestershire Royal Hospital, Gloucester, United Kingdom

[5] Department of Anaesthetics, Cairns Hospital, Cairns, Queensland, Australia

[6] Aboriginal and Torres Strait Islander Health, Torres and Cape Hospital and Health Service, Cooktown, Queensland, Australia

* Corresponding author:

Email: jhanson@kirby.unsw.edu.au.

Short title: The utility of predictive scores in Indigenous Australians with sepsis

Abstract

Background: Indigenous Australians suffer a disproportionate burden of sepsis, however, the performance of scoring systems that predict mortality in Indigenous patients with critical illness is incompletely defined.

Materials and methods: The study was performed at an Australian tertiary-referral hospital between January 2014 and June 2017, and enrolled consecutive Indigenous and non-Indigenous adults admitted to ICU with sepsis. The ability of the ANZROD, APACHE-II, APACHE-III, SAPS-II, SOFA and qSOFA scores to predict death before ICU discharge in the two populations was compared.

Results: There were 442 individuals enrolled in the study, 145 (33%) identified as Indigenous. Indigenous patients were younger than non-Indigenous patients (median (interquartile range (IQR) 53 (43-60) versus 65 (52-73) years, p=0.0001) and comorbidity was more common (118/145 (81%) versus 204/297 (69%), p=0.005). Comorbidities that were more common in the Indigenous patients included diabetes mellitus (84/145 (58%) versus 67/297 (23%), p<0.0001), renal disease (56/145 (39%) versus 29/297 (10%), p<0.0001) and cardiovascular disease (58/145 (40%) versus 83/297 (28%), p=0.01). The use of supportive care (including vasopressors, mechanical ventilation and renal replacement therapy) was similar in Indigenous and non-Indigenous patients, and the two populations had an overall case-fatality rate that was comparable (17/145 (12%) and 38/297 (13%) (p=0.75)), although Indigenous patients died at a younger age (median (IQR): 54 (50-60) versus 70 (61-76) years, p=0.0001). There was no significant difference in the ability of any the scores to predict mortality in the two populations.

Conclusions: Although the crude case-fatality rates of Indigenous and non-Indigenous Australians admitted to ICU with sepsis is comparable, Indigenous patients die at a much younger age. Despite this, the ability of commonly used scoring systems to predict outcome in Indigenous Australians is similar to that of non-Indigenous Australians, supporting their use in

ICUs with a significant Indigenous patient population and in clinical trials that enrol Indigenous Australians.

Introduction

Globally, sepsis is estimated to kill 5.3 million people every year, predominantly in low- and middle income countries (1). Even in the well-resourced Australian health system, sepsis kills over 5000 people annually (2, 3). However, the case-fatality rate is not uniform across Australia (4). This can be partly explained by differences in the complexity of patient care at different sites, but other factors including patient age and demographics, the geographical location of the Intensive Care Unit (ICU), and the local prevalence of different pathogens also contribute (4).

In an effort to ensure that all Australians admitted to ICU are receiving high-quality care, predictive scoring systems are used to measure patients' disease severity and determine their expected outcomes (5-9). While these scores have limited utility in the management of individual patients, they can be used by institutions to benchmark ICU performance. They are also important in clinical trials where they can be used to help evaluate interventions by providing a measure of study patients' disease severity. However, these scoring systems are most frequently derived and validated in metropolitan referral centres in high-income settings, their predictive ability may differ in other patient populations (10, 11).

Indigenous Australians are disproportionately represented in Australian ICUs and sepsis is one of the most common indications for admission (12). Several studies have examined the characteristics of undifferentiated cohorts of Indigenous Australians admitted to ICU, and have identified that they are younger, have greater comorbidity and live more frequently in remote locations (12-15). While, the overall case-fatality rate of Indigenous Australians is similar to that of non-Indigenous Australians in these series, there has been limited examination of the comparative performance of predictive scoring systems in Indigenous patients (12-15). This is

important, because in some parts of the country, Indigenous Australians represent a significant proportion of ICU admissions (13, 14).

This study was performed to determine the applicability of commonly used predictive scoring systems in Indigenous Australians admitted to ICU with sepsis. It was hoped that the study might validate the use of these scoring systems in both Aboriginal and Torres Strait Islander Australians, which would support their use in future clinical sepsis trials enrolling Indigenous patients. It would also provide justification for their use in benchmarking the performance of ICUs with a greater proportion of Indigenous patients (16).

Materials and methods

This retrospective study used data collected in the ICU of Cairns Hospital, the only ICU in the Far North Queensland (FNQ) region. FNQ's population of 279,354 is dispersed across an area of 204,255 km^2; approximately 17% of whom identify as Indigenous Australians (17).

Consecutive adults (≥ 18 years) admitted between 1 January 2014 and 30 June 2017 to the ICU with a primary admission diagnosis of sepsis (APACHE III-J diagnostic codes 501-504) were eligible for the study. Demographic, clinical and laboratory data were collected and correlated with the patients' clinical course. Comorbidities were said to be present if documented in the medical record (MetaVision®). Hazardous alcohol consumption was defined as regular consumption of greater than 4 standard drinks/day. The data were collected by 4 members of the research team (SH SS TG JB) who met regularly to confirm uniformity of documentation. If patients were admitted to ICU more than once during the study period, only their first admission was included in the analysis.

All individuals receiving care in Queensland's public health system, are asked whether they identify as an Aboriginal Australian, a Torres Strait Islander Australian, both or neither. Commonly used disease severity prediction scores including the Australia and New Zealand Risk Of Death (ANZROD) score (5), the Acute Physiology, Age, Chronic Health Evaluation-II and III (APACHE-II and APACHE-III) scores (18), the Simplified Acute Physiology Score (SAPS) II score (19) and the quick SOFA (qSOFA) score (20) were calculated using variables collected at the time of ICU admission. The Sequential Organ Failure Assessment (SOFA) score was calculated using the worst values recorded in the first 24 hours of the ICU admission (9). The scores' ability to predict both death prior to ICU discharge and at 90 days was determined.

Statistical analysis

Data were de-identified, entered in an electronic database (Microsoft Excel) and analysed using statistical software (Stata version 14.2). Groups were compared using the Kruskal-Wallis, Chi-square test or Fisher's exact test where appropriate. Multivariate analysis was performed using backwards stepwise logistic regression. The Indigenous population was further examined by dividing the Indigenous population into those who identified as Aboriginal Australians and those who identified as Torres Strait Islander Australians. Those who identified as both, were not included in analyses comparing the two Indigenous populations.

The ability of the scores to predict death were determined by measuring the area under receiver operator characteristic (AUROC) curve; the optimal cut-off for the tests for the different populations were determined using Liu's method (21).

Ethics approval

Ethics approval was obtained from the Far North Queensland Human Research Ethics Committee (HREC/17/QCH/93/AMO2). As the data were retrospective and de-identified, the Committee waived the requirement for informed consent.

Results

There were 442 individuals admitted to ICU for sepsis, on 500 occasions, during the study period. Their median (interquartile range (IQR)) age at their first presentation was 59 (48-70) years, 238 (54%) were male. Of the 442 patients, 145 (33%) identified as Indigenous Australians, 94 (65%) identified as Aboriginal, 36 (25%) identified as Torres Strait Islanders, while 15 (10%) identified as both. Among the 442 patients, 416 (94%) were FNQ residents; Indigenous patients were more likely to reside in a rural or remote location than non-Indigenous patients (88/144 (61%) versus 128/272 (47%), p=0.006).

Patient characteristics

Indigenous patients were younger than non-Indigenous patients (median (IQR) 53 (43-60) versus 65 (52-73) years, p=0.0001) and were more likely to have a significant comorbidity (118/145 (81%) versus 204/297 (69%), p=0.005).

Indigenous patients were more likely to have diabetes mellitus than non-Indigenous patients (odds ratio (OR): 4.7, 95% confidence interval (CI): 3.1-7.2), which contributed to a significantly greater burden of cardiovascular disease and renal disease (table 1). Diabetes was more common in Torres Strait Islanders than Aboriginal Australians (27/36 (75%) versus 48/94 (51%), p= 0.01). No fewer than 32/145 (22%) Indigenous patients had a history of sepsis (prior hospitalisation with APACHE III-J diagnostic codes 501-504).

While Indigenous patients, as a group, were more likely to have a history of hazardous alcohol or tobacco use, hazardous alcohol use was more common in the Aboriginal patients than in Torres Strait Islanders (49/94 (52%) versus 7/36 (9%) (p=0.001). The difference in smoking rates between Aboriginal and Torres Strait Islanders failed to reach statistical significance (63/94 (67%) versus 18/36 (50%), p=0.07). Other demographic characteristics of the cohort and their comorbidities - stratified by Indigenous status - are presented in table 1.

Nature of the sepsis

The commonest presumed source of sepsis was the respiratory tract, although skin and soft tissue infections (SSTI) were also common and occurred more frequently in Indigenous patients, particularly those with diabetes (31/41 (76%) Indigenous patients with a SSTI were diabetic, compared with 53/104 (51%) with another source, p=0.007). *Staphylococcus aureus* was the pathogen isolated most commonly and was responsible for 66 (15%) admissions; 13 (18%) of the isolates were methicillin resistant. In general, however, drug resistant pathogens and "tropical" pathogens were relatively uncommon (table 2).

Clinical and laboratory findings at presentation

Renal impairment and metabolic acidosis were more common among Indigenous patients, but other laboratory findings in the Indigenous and non-Indigenous patients were similar (table 3).

Supportive care

The supportive care provided to the Indigenous and non-Indigenous patients was very similar. While a greater proportion of Indigenous patients required renal replacement therapy (RRT), this did not reach statistical significance (19/145 (13%) versus 27/297 (9%), p=0.20) (table 4).

Case-fatality rate

There were 55 deaths in the cohort prior to ICU discharge: 17/145 (12%) Indigenous patients versus 38/297 (13%) non-Indigenous patients, p=0.75. There were 14 (15%) deaths among the 94 Aboriginal Australians and 2 (6%) among the 36 Torres Strait Islander Australians (p=0.23). There was one (7%) death among the 15 who identified as both Aboriginal and Torres Strait Islander Australians.

Patients that died before ICU discharge were older than those that survived (median (IQR): 62 (54-73) versus 59 (46-69 years), p=0.01) and more likely to have a significant comorbidity (47/55 (85%) versus 275/387 (71%), p=0.03). Among the 442 FNQ residents, 29/50 (58%) living in a rural or remote location died compared with 187/366 (51%), with an urban address (p=0.36). In a multivariate analysis model which included age, Indigenous status, significant comorbidity and remote or rural residence, only age had a statistically significant association with death (OR: 1.03 (95%CI): 1.01-1.05), p=0.007).

After 90 days, 93/442 (21%) had died. Death at 90 days was linked to age (p=0.0001), but not to Indigenous status (p=0.26) or rural/remote residence (p=0.29). Indigenous patients, however, died at a younger age than non-Indigenous patients (median (IQR): 56 (52-62) versus 68 (61-76) years, p=0.0001).

Disease severity scores and their ability to predict death

The severity scores of the Indigenous and non-Indigenous patients were similar (table 5). There was no statistically significant difference in the ability of the various severity scores to predict death in Indigenous and non-Indigenous patients (tables 6 and 7). Among Indigenous patients, the ANZROD and APACHE-III scores had the highest AUROC curve (0.85 (95% CI: 0.77-0.92 and 0.84 (95% CI: 0.76-0.92), although these values were only statistically superior to that of the qSOFA scores (tables 6 and 7). The very low case-fatality rate in the Torres Strait

Islanders and relatively small sample size precluded meaningful comparison of the relative performance of the prediction scores in Aboriginal and Torres Strait Islanders.

Discussion

In this cohort of ICU patients with sepsis there were significant differences in the age, burden of comorbidities and source of infection between Indigenous and non-Indigenous individuals. However, the ability of commonly used disease severity scoring systems to predict mortality in the two populations was similar.

FNQ, in tropical Australia, shares a border with Papua New Guinea and has a unique blend of infectious diseases; it has the country's highest incidence of leptospirosis and an increasing incidence of melioidosis and rickettsial disease (22-24). The rates of methicillin-resistance in *S. aureus* isolates are among the highest reported in the country, while antibiotic resistance in other common pathogens is also increasing (25, 26). The population is widely dispersed across a large geographical area and there are very limited specialist services outside the administrative hub of Cairns. It is a region which contains 3 of the 10 most socio-economically disadvantaged local government areas in the country, all 3 are communities with a predominantly Indigenous population (27). It is the only part of Australia which has the homelands of both Aboriginal and Torres Strait Islander Australians, peoples that are frequently conflated but who are ethnologically quite distinct. All these factors might be expected to have implications for local patterns of sepsis, its presentation and its outcomes (16, 28-30). However, while Indigenous patients were younger, more likely to be live in a rural/remote location and have a significant comorbidity than non-Indigenous patients, the severity of their sepsis – as determined by commonly used predictive scoring systems was similar to that of non-Indigenous patients.

Although Indigenous Australians bear a greater burden of sepsis, there are surprisingly few studies that examine sepsis in the Indigenous population systematically. The demography and comorbidities in our series are very similar to that of a prospective study of sepsis in the Northern Territory, which also included patients that were not admitted to the ICU (16). That study identified that Indigenous Australians were over-represented in the cohort, suffered disproportionately from diabetes and renal disease, and reported higher rates of smoking and hazardous alcohol use. Although APACHE-II and SOFA scores were collected in the study, the comparability of their performance in Indigenous and non-Indigenous populations was not presented.

While there are relatively few published data examining the ICU care of Australian Indigenous patients with sepsis specifically, there are several studies that have examined the clinical characteristics and outcomes of Indigenous patients admitted to ICU (12-15). These studies are strikingly similar and show that Indigenous patients with critical illness are younger, have greater comorbidity and are more frequently admitted from remote locations than non-Indigenous patients. These studies also universally show that there is no difference in the proportion of Indigenous and non-Indigenous patients who die in the ICU. Whilst some authors have suggested that Indigenous deaths in ICU are therefore "healthcare preventable" (14), this represents a narrow view of healthcare, de-emphasising the primary holistic care that should prevent the hospitalisation in the first place. Whilst other authors have noted that there is "no mortality gap" between Indigenous and non-Indigenous Australians admitted to ICU (12), this overlooks the fact the Indigenous patients who are admitted to ICU are younger and are dying at a younger age. In an ICU series from the Northern Territory, 7% of both Indigenous and non-Indigenous patients admitted to the ICU died, but the Indigenous patients who died were much younger than their non-Indigenous counterparts (mean age of 45 versus 56) (13). This pattern was seen in our series: although the proportion of Indigenous patients and non-

Indigenous patients dying before ICU discharge was similar, the median age of death of Indigenous patients (56 years) was 12 years lower than that of the non-Indigenous patients. To close this gap, we need evidence-based strategies to address the unique challenges faced by Indigenous Australians in the country's health system. Our study shows that predictive scoring systems are a valid way of measuring disease severity among Indigenous Australians with sepsis and can therefore be used to compare the efficacy of interventions with confidence.

Our study has many limitations. As a single centre trial, it reflects only the experience of a unique part of Australia; the applicability of the results to the broader Indigenous patient population requires validation, although the similarity between the Indigenous population seen in this study and those in other locations is striking (12-16). The study was retrospective and examined only patients admitted to ICU and will therefore underestimate the true sepsis burden (31).

Clearly there is still much to do to address the disparity in health outcomes between Indigenous and non-Indigenous Australians (30). Once Indigenous Australians with sepsis enter ICU, they receive high quality care, but they are still dying at a much younger age than their non-Indigenous counterparts. Interventions at a community level including efforts to facilitate access to care, reduce crowding, enhance health literacy, improve sanitation and ensure appropriate nutrition are likely to be most helpful (32). At a primary health level optimising management of conditions like diabetes that predispose to sepsis and ensuring comprehensive vaccination, particularly for those at high risk are also likely to assist. Expanded programmes to assist with smoking cessation and encourage alcohol moderation are also essential (33, 34). However, even with optimal preventative interventions, patients will still require ICU care for sepsis. This study suggests that standard predictive scores predict outcomes in Indigenous

patients as well as they do in non-Indigenous patients and therefore may be used in future studies to examine strategies to enhance the care of all Australians

Acknowledgements

The authors would like to acknowledge all the health workers who were involved in the care of the patients.

References

1. Fleischmann C, Scherag A, Adhikari NK, Hartog CS, Tsaganos T, Schlattmann P, et al. Assessment of Global Incidence and Mortality of Hospital-treated Sepsis. Current Estimates and Limitations. Am J Respir Crit Care Med. 2016;193(3):259-72.
2. Sepsis epidemiology: Australian Sepsis Network; 2019 [Available from: https://www.australiansepsisnetwork.net.au/healthcare-providers/sepsis-epidemiology.
3. Kaukonen KM, Bailey M, Suzuki S, Pilcher D, Bellomo R. Mortality related to severe sepsis and septic shock among critically ill patients in Australia and New Zealand, 2000-2012. JAMA. 2014;311(13):1308-16.
4. Arise, Committee AAM. The outcome of patients with sepsis and septic shock presenting to emergency departments in Australia and New Zealand. Crit Care Resusc. 2007;9(1):8-18.
5. Paul E, Bailey M, Pilcher D. Risk prediction of hospital mortality for adult patients admitted to Australian and New Zealand intensive care units: development and validation of the Australian and New Zealand Risk of Death model. J Crit Care. 2013;28(6):935-41.
6. Cook DA. Performance of APACHE III models in an Australian ICU. Chest. 2000;118(6):1732-8.
7. Moreno RP, Metnitz PG, Almeida E, Jordan B, Bauer P, Campos RA, et al. SAPS 3--From evaluation of the patient to evaluation of the intensive care unit. Part 2: Development of a prognostic model for hospital mortality at ICU admission. Intensive Care Med. 2005;31(10):1345-55.
8. Higgins TL, Teres D, Copes WS, Nathanson BH, Stark M, Kramer AA. Assessing contemporary intensive care unit outcome: an updated Mortality Probability Admission Model (MPM0-III). Crit Care Med. 2007;35(3):827-35.
9. Vincent JL, Moreno R, Takala J, Willatts S, De Mendonca A, Bruining H, et al. The SOFA (Sepsis-related Organ Failure Assessment) score to describe organ dysfunction/failure. On behalf of the Working Group on Sepsis-Related Problems of the European Society of Intensive Care Medicine. Intensive Care Med. 1996;22(7):707-10.

10. Bouch DC, Thompson JP. Severity scoring systems in the critically ill. Continuing Education in Anaesthesia Critical Care & Pain. 2008;8(5):181-5.
11. Moreno RP. Outcome prediction in intensive care: why we need to reinvent the wheel. Curr Opin Crit Care. 2008;14(5):483-4.
12. Secombe P, Brown A, McAnulty G, Pilcher D. Aboriginal and Torres Strait Islander patients requiring critical care: characteristics, resource use, and outcomes. Crit Care Resusc. 2019;21(3):200-11.
13. Stephens D. Critical illness and its impact on the Aboriginal people of the top end of the Northern Territory, Australia. Anaesth Intensive Care. 2003;31(3):294-9.
14. Trout MI, Henson G, Senthuran S. Characteristics and outcomes of critically ill Aboriginal and/or Torres Strait Islander patients in North Queensland. Anaesth Intensive Care. 2015;43(2):216-23.
15. Ho KM, Finn J, Dobb GJ, Webb SA. The outcome of critically ill Indigenous patients. Med J Aust. 2006;184(10):496-9.
16. Davis JS, Cheng AC, McMillan M, Humphrey AB, Stephens DP, Anstey NM. Sepsis in the tropical Top End of Australia's Northern Territory: disease burden and impact on Indigenous Australians. Med J Aust. 2011;194(10):519-24.
17. Lee A, Hanson J, Fox P, Spice G, Russell D, Boyd P. A decentralised, multidisciplinary model of care facilitates treatment of hepatitis C in regional Australia. J Virus Erad. 2018;4(3):160-4.
18. Knaus WA, Wagner DP, Draper EA, Zimmerman JE, Bergner M, Bastos PG, et al. The APACHE III prognostic system. Risk prediction of hospital mortality for critically ill hospitalized adults. Chest. 1991;100(6):1619-36.
19. Le Gall JR, Lemeshow S, Saulnier F. A new Simplified Acute Physiology Score (SAPS II) based on a European/North American multicenter study. JAMA. 1993;270(24):2957-63.
20. Seymour CW, Liu VX, Iwashyna TJ, Brunkhorst FM, Rea TD, Scherag A, et al. Assessment of Clinical Criteria for Sepsis: For the Third International Consensus Definitions for Sepsis and Septic Shock (Sepsis-3). JAMA. 2016;315(8):762-74.
21. Liu X. Classification accuracy and cut point selection. Stat Med. 2012;31(23):2676-86.
22. Stewart AGA, Smith S, Binotto E, McBride WJH, Hanson J. The epidemiology and clinical features of rickettsial diseases in North Queensland, Australia: Implications for patient identification and management. PLoS Negl Trop Dis. 2019;13(7):e0007583.
23. Stewart JD, Smith S, Binotto E, McBride WJ, Currie BJ, Hanson J. The epidemiology and clinical features of melioidosis in Far North Queensland: Implications for patient management. PLoS Negl Trop Dis. 2017;11(3):e0005411.
24. Smith S, Kennedy BJ, Dermedgoglou A, Poulgrain SS, Paavola MP, Minto TL, et al. A simple score to predict severe leptospirosis. PLoS Negl Trop Dis. 2019;13(2):e0007205.
25. Guthridge I, Smith S, Horne P, Hanson J. Increasing prevalence of methicillin-resistant Staphylococcus aureus in remote Australian communities: implications for patients and clinicians. Pathology. 2019;51(4):428-31.
26. Nofz L, Koppen J, De Alwis N, Smith S, Hanson J. The microbiology of ear cultures in a high-burden setting in tropical Australia: Implications for clinicians. Clin Otolaryngol. 2019;44(6):1195-200.
27. 2071.0 - Census of Population and Housing: Reflecting Australia - Stories from the Census, 2016 Canberra, Australia: Australian Bureau of Statistics; 2018 [Available from: https://www.abs.gov.au/ausstats/abs@.nsf/Lookup/by%20Subject/2071.0~2016~Main%20Features~Socio-Economic%20Advantage%20and%20Disadvantage~123.

28. Tong SY, van Hal SJ, Einsiedel L, Currie BJ, Turnidge JD, Australian New Zealand Cooperative on Outcomes in Staphylococcal S. Impact of ethnicity and socio-economic status on Staphylococcus aureus bacteremia incidence and mortality: a heavy burden in Indigenous Australians. BMC Infect Dis. 2012;12:249.

29. Tosas Auguet O, Betley JR, Stabler RA, Patel A, Ioannou A, Marbach H, et al. Evidence for Community Transmission of Community-Associated but Not Health-Care-Associated Methicillin-Resistant Staphylococcus Aureus Strains Linked to Social and Material Deprivation: Spatial Analysis of Cross-sectional Data. PLoS Med. 2016;13(1):e1001944.

30. The health and welfare of Australia's Aboriginal and Torres Strait Islander peoples. Cat. no. IHW 147. Canberra, Australia: Australian Institute of Health and Welfare; 2015.

31. Jolley RJ, Sawka KJ, Yergens DW, Quan H, Jette N, Doig CJ. Validity of administrative data in recording sepsis: a systematic review. Crit Care. 2015;19:139.

32. Aboriginal and Torres Strait Islander Health Performance Framework (HPF) report 2017 Canberra: Australian Institute of Health and Welfare; 2017 [Cat. no: IHW 194:[Available from: https://www.aihw.gov.au/reports/indigenous-australians/health-performance-framework/contents/overview.

33. Huttunen R, Heikkinen T, Syrjanen J. Smoking and the outcome of infection. J Intern Med. 2011;269(3):258-69.

34. Trevejo-Nunez G, Kolls JK, de Wit M. Alcohol Use As a Risk Factor in Infections and Healing: A Clinician's Perspective. Alcohol Res. 2015;37(2):177-84.

Table 1 Demographic characteristics and comorbidities of the cohort, stratified by Indigenous status

Variable	Indigenous n=145	Non-Indigenous n=297	p
Age	53 (43-60)	65 (52-73)	0.0001
Male gender	68 (47%)	170 (57%)	0.04
Residence in a remote location [a]	88/144 (61%)	128/272 (47%)	0.006
Inter-hospital transfer	56 (39%)	115 (39%)	0.98
Admitted from Emergency Department	44 (30%)	95 (32%)	0.73
Admitted from a nursing home	0	0	-
Planned admission after surgery	3 (2%)	2 (1%)	0.34
Admitted from hospital ward	42 (29%)	83 (28%)	0.82
Admission at night	94 (65%)	182 (61%)	0.47
Hazardous alcohol consumption	58 (40%)	53 (18%)	<0.0001
Cigarette smoker	88 (61%)	134 (45%)	0.002
History of sepsis	32 (22%)	30 (10%)	0.001
History of cardiovascular disease	58 (40%)	83 (28%)	0.01
History of respiratory disease	31 (21%)	49 (17%)	0.21
History of renal disease	56 (39%)	29 (10%)	<0.0001
History of haematological disease or malignancy	7 (5%)	29 (10%)	0.10
History of liver disease	18 (12%)	22 (7%)	0.09
History of diabetes mellitus	84 (58%)	67 (23%)	<0.0001
History of metastatic cancer	6 (4%)	29 (10%)	0.04
Immunocompromised	9 (6%)	52 (18%)	0.001
Significant comorbidity	118 (81%)	204 (69%)	0.005
Body mass index	27 (22-33)	28 (24-33)	0.07

Absolute numbers (%) or median (interquartile range) are presented.
[a] Only includes the 416 Far North Queensland residents.

Table 2 Source and aetiology of the sepsis, stratified by Indigenous status

Variable	Indigenous n=145	Non- Indigenous n=297	p
Respiratory source	51 (35%)	97 (33%)	0.60
Genitourinary source	32 (22%)	64 (22%)	0.90
Bone/joint source	7 (5%)	6 (2%)	0.13
Central nervous system source	0 (0%)	6 (2%)	0.18
Skin/soft tissue source	41 (28%)	43 (14%)	0.0001
Abdominal source	13 (9%)	43 (14%)	0.10
Other source	10 (7%)	47 (16%)	0.01
Bacterial infection	103(71%)	194 (65%)	0.23
Gram negative bacteria	63 (43%)	131 (44%)	0.90
Gram positive bacteria	55 (38%)	84 (28%)	0.04
Fungal infection	7 (5%)	9 (3%)	0.42
Viral infection	10 (7%)	22 (7%)	0.85
Drug resistant organism	5 (3%)	11 (4%)	1.0
Bacteraemia	56 (39%)	128 (43%)	0.37
Polymicrobial infection	34 (23%)	46 (15%)	0.04
Escherichia coli	15 (10%)	44 (15%)	0.20
Staphylococcus aureus	26 (18%)	40 (13%)	0.22
Methicillin-resistant *S. aureus*	7 (5%)	6 (2%)	0.13
Pseudomonas aeruginosa	15 (10%)	28 (9%)	0.76
Klebsiella pneumoniae	9 (6%)	15 (5%)	0.66
Influenza A	5 (3%)	13 (4%)	0.80
Burkholderia pseudomallei	8 (6%)	8 (3%)	0.17
Streptococcus pyogenes	16 (11%)	9 (3%)	0.001
Leptospirosis	0	11 (4%)	0.02
Streptococcus pneumoniae	6 (4%)	10 (3%)	0.79
Pneumocystis jirovecii	2 (1%)	4 (1%)	1.0
Mycobacterium tuberculosis	0	1 (0.3%)	1
Plasmodium falciparum	0	1 (0.3%)	1
Rickettsia australis	1 (0.7%)	0	0.33
Vibrio vulnificus	1 (0.7%)	0	0.33

Absolute numbers (%) presented.

Table 3 Clinical and laboratory findings at presentation, stratified by Indigenous status

Variable	Indigenous n=145	Non- Indigenous n=297	p
Heart rate (beats/min)	99 (89-118)	97 (81-114)	0.28
Systolic blood pressure (mmHg)	107 (94-118)	106 (94-122)	0.42
Mean arterial pressure (mmHg)	73 (66-82)	72 (65-83)	0.50
Temperature (°C)	36.8 (36.5-37.4)	36.9 (36.5-37.3)	0.53
Respiratory rate (breaths/min)	20 (17-26)	20 (16-25)	0.99
Glasgow Coma Score	15 (14-15)	15 (14-15)	0.98
Glucose (mmol/L)	6.9 (5.0-9.4)	6.6 (5.4-8.5)	0.96
Lactate (mmol/L)	1.8 (1.1-3.0)	1.7 (1.1-2.8)	0.29
pH	7.36 (7.24-7.42)	7.37 (7.30-7.43)	0.13
PaO_2/FiO_2	291 (194-398)	269 (158-375)	0.02
$PaCO_2$ (mmHg)	34 (27-39)	34 (29-42)	0.0496
Bicarbonate (mmol/L)	18 (15-21)	20 (17-23)	0.001
Base excess (mmol/L)	-6.2 (-10.9 to -2.8)	-4.5 (-8.4 to -2.2)	0.004
Anion gap	9 (7-12)	9 (7-12)	0.44
Haemoglobin (g/dL)	100 (87-122)	111 (93-124)	0.007
White cell count ($x10^9$/L)	13.8 (9.3-21.9)	13.8 (7.9-22.2)	0.67
Neutrophils ($x10^9$/L)	10.7 (7.1-19.2)	11.6 (6.2-19.4)	0.80
Eosinophils ($x10^9$/L)	0 (0-0.1)	0(0-0)	0.001
Platelets ($x10^9$/L)	170 (111-257)	1650(101-236)	0.17
C-reactive protein (mg/L))	165 (67-295)	172 (85-286)	0.57
Troponin I (ng/mL)	0.07 (0.04-0.39)	0.11 (0.04-0.43)	0.85
Prothrombin (seconds)	16 (14-21)	16 (14-18)	0.07
APTT (seconds)	39 (34-47)	36 (31-40)	0.0001
INR	1.5 (1.3-1.9)	1.4 (1.3-1.6)	0.03
Fibrinogen (g/L)	6.2 (4.6-8.3)	6.6 (4.9-8.2)	0.35
Total Bilirubin (µmol/L)	18 (12-31)	20 (13-31)	0.31
Conjugated bilirubin (µmol/L)	8 (4-19)	7 (4-15)	0.43
Albumin (g/L)	24 (20-27)	25 (22-29)	0.007
Protein (g/L)	58 (52-65)	52 (48-60)	0.0001
AST (IU/mL)	45 (21-104)	52 (27-106)	0.12
ALT (IU/mL)	25 (12-42)	35 (20-65)	0.0001
GGT (IU/mL)	41 (22-65)	51 (26-94)	0.01
ALP (IU/mL)	91 (69-134)	82 (57-119)	0.01
LDH (IU/mL)	333 (248-482)	329 (239-439)	0.43
Sodium (mmol/L)	133 (131-137)	135 (133-138)	0.0004
Potassium (mmol/L)	4.1 (3.6-4.8)	4.0 (3.7-4.5)	0.31
Chloride (mmol/L)	104 (99-109)	104 (100-108)	0.92
Creatinine (µmol/L)	162 (87-403)	118 (75-190)	0.0003
eGFR ($ml/min/1.73m^2$)	29 (11-65)	49 (24-80)	0.0002
Calcium (mmol/L)	2.2 (2.1-2.3)	2.2 (2.1-2.3)	0.99
Magnesium (mmol/L)	0.73 (0.66-0.87)	0.75 (0.65-0.88)	0.48
Phosphate (mmol/L)	1.5 (1.0-2.0)	1.1 (0.9-1.5)	0.0001

Median (Interquartile range) presented. PaO_2/FiO_2: ratio of arterial oxygen partial pressure (PaO2 in mmHg) to fractional inspired oxygen. $PaCO_2$: arterial carbon dioxide partial pressure. APTT: activated partial thromboplastin time; INR: International normalised ratio; AST: aspartate aminotransferase; ALT: alanine aminotransferase; GGT: Gamma-glutamyl transferase; ALP: alkaline phosphatase; LDH: lactate dehydrogenase; eGFR: estimated glomerular filtration rate.

Table 4 Supportive care delivered to the cohort, stratified by Indigenous status

Variable	Indigenous n=145	Non- Indigenous n=297	p
Vasopressors on admission	102 (70%)	204 (69%)	0.72
Number of vasopressors required	1 (1-1)	1 (0-1)	0.89
Antibiotics administered on admission	142 (98%)	288 (97%)	0.56
Endotracheal intubation	28 (19%)	64 (22%)	0.59
Minute ventilation	7.3 (5.8 - 8.8)	7.7 (6.6 – 9.0)	0.30
PICC line	40 (28%)	89 (30%)	0.61
Central venous line	61 (42%)	121 (41%)	0.79
Arterial line	122 (84%)	246 (83%)	0.73
Nasogastric feeding	28 (19%)	63 (21%)	0.64
Indwelling urinary catheter	117 (81%)	252 (85%)	0.27
Renal replacement therapy	19 (13%)	27 (9%)	0.20

Absolute numbers (%) and median (interquartile range) presented. PICC: Peripherally inserted central catheter

Table 5 Severity score values on admission to ICU, stratified by Indigenous status

Variable	number	Indigenous n=145	Non- Indigenous n=297	p
qSOFA	348	1 (1-2)	1 (1-2)	0.83
SOFA	378	9 (6-11)	8 (6-11)	0.20
ANZROD	430	0.24 (0.10-0.44)	0.23 (0.10-0.46)	0.86
APACHE-II	430	21 (15-26)	20 (15-26)	0.65
APACHE-III	430	70 (52-87)	69 (53-87)	0.87
SAPS-II	413	35 (25-51)	38 (28-58)	0.12

Median (interquartile range) presented. qSOFA: quick SOFA score; SOFA: Sequential Organ Failure Assessment score; ANZROD: Australia and New Zealand Risk Of Death score; APACHE-II: Acute Physiology, Age, Chronic Health Evaluation-II score; and APACHE-III: Acute Physiology, Age, Chronic Health Evaluation-III score; SAPS-II: Simplified Acute Physiology Score.

Table 6 Performance of the severity scores in predicting death before ICU discharge, stratified by Indigenous status

Severity score	Number of Indigenous patients in whom the score could be calculated	AUROC (95% CI)	Optimal cut-off in Indigenous patients	Number of Non- Indigenous patients in whom the score could be calculated	AUROC (95% CI)	Optimal cut-off in Non-Indigenous patients	p
qSOFA	116	0.71 (0.57-0.84)	2	232	0.58 (0.46-0.70)	2	0.17
SOFA	120	0.75 (0.64-0.87)	10	258	0.72 (0.63-0.80)	10	0.62
ANZROD	138	0.85 (0.77-0.92)	0.33	292	0.79 (0.71-0.87)	0.35	0.32
APACHE-II	138	0.78 (0.64-0.91)	30	292	0.76 (0.67-0.84)	27	0.61
APACHE-III	138	0.84 (0.76-0.92)	82	292	0.79 (0.71-0.87)	88	0.37
SAPS-II	136	0.80 (0.70-0.90)	46	277	0.85 (0.78-0.92)	66	0.40

qSOFA: quick SOFA score; SOFA: Sequential Organ Failure Assessment score; ANZROD: Australia and New Zealand Risk Of Death score; APACHE-II: Acute Physiology, Age, Chronic Health Evaluation-II score; and APACHE-III: Acute Physiology, Age, Chronic Health Evaluation-III score; SAPS-II: Simplified Acute Physiology Score.

Table 7 Performance of the severity scores in predicting 90-day mortality, stratified by Indigenous status

Severity score	Number of Indigenous patients in whom the score could be calculated	AUROC (95% CI)	Optimal cut-off in Indigenous patients	Number of Non-Indigenous patients in whom the score could be calculated	Non-Indigenous n=297	Optimal cut-off in Non-Indigenous patients	p
qSOFA	116	0.66 (0.53-0.79)	2	232	0.59 (0.50-0.68)	2	0.42
SOFA	120	0.75 (0.64-0.85)	11	258	0.66 (0.58-0.74)	9	0.22
ANZROD	138	0.85 (0.79-0.92)	0.33	292	0.78 (0.71-0.84)	0.29	0.09
APACHE-II	138	0.74 (0.62-0.85)	30	292	0.72 (0.65-0.79)	21	0.78
APACHE-III	138	0.85 (0.78-0.92)	73	292	0.77 (0.71-0.84)	75	0.13
SAPS-II	136	0.78 (0.68-0.88)	36	277	0.75 (0.69-0.82)	39	0.66

qSOFA: quick SOFA score; SOFA: Sequential Organ Failure Assessment score; ANZROD: Australia and New Zealand Risk Of Death score; APACHE-II: Acute Physiology, Age, Chronic Health Evaluation-II score; and APACHE-III: Acute Physiology, Age, Chronic Health Evaluation-III score; SAPS-II: Simplified Acute Physiology Score.

Dataset

Click here to access/download
Supporting Information
Final dataset for publication.xlsx
