# Supplementary material for: The applicability of commonly used predictive scoring systems in Indigenous Australians with sepsis: An observational study
Source: PLoS One. 2020 Jul 22;15(7):e0236339. doi: 10.1371/journal.pone.0236339 (PMC7375531; doi:10.1371/journal.pone.0236339)
Supplement: Attachment — Submitted filename: PLOS review PONE-D-20-14432 20200609.docx [file pone.0236339.s003.docx]

PLOS-ONE PONE-D-20-14432 reviewer notes

Abstract:

Materials and methods: Cairns is referred to as a “tertiary-referral Hospital”.

According to QH documentation and the Service’s homepage Cairns Hospital is not a referral hospital (the homepage states: “main referring hospitals: The Townsville Hospital 344 km, Royal Brisbane and Women’s Hospital 1680 km).

So I suggest to use the term Base Hospital or a similar term, or to refer to the Clinical Services Capability Framework of Queensland Health 9CSCF)

Results: figures as expected

Conclusions: “supporting their use”

I think this needs further clarification!

Whilst not discussed in the paper, someone just reading the abstract may think the scores can be used to tailor treatment / intervention for the individual patient. To my opinion none of the scores showed an AUROC sufficient to be used as a prognostic tool for an individual for death, so to decide about how far to escalate the therapeutic effort for the individual patient and when to stop escalating therapy (for example, whom to ventilate, where to use ECMO, CHHV, increase inotropes). The closest score is APACHE-III with an AUROC of 0.84. This also is not surprising and would not need a score – it probably is just common sense that the more interventions you need to keep a patient alive, the more likely they are to die.

Of course, for the purpose of comparing patients for benchmarks, activity comparison and funding, studies, the scoring systems definitely are useful, as stated in the discussion P 12 266-267.

I would suggest to change the sentence in the abstract to “… supporting their use to compare the efficacy of interventions …”

P4-5 Introduction

There is no definition of sepsis provided.

P8 Clinical and laboratory findings

Metabolic acidosis (Bicarbonate 0.001, BE 0.004) must be more common if renal impairment is more common Crea/ eGFR P << 0.001), so Metabolic Acidosis is not an independent variable, may be worth to comment.

Also, probably worth to comment: no significant abnormalities in LFTs, Platelet count (there is a typo in the table on P 18 that needs correction), Albumin for both the Indigenous and non-Indigenous patient groups.
