# Supplementary material for: The applicability of commonly used predictive scoring systems in Indigenous Australians with sepsis: An observational study
Source: PLoS One. 2020 Jul 22;15(7):e0236339. doi: 10.1371/journal.pone.0236339 (PMC7375531; doi:10.1371/journal.pone.0236339)
Supplement: Attachment — Submitted filename: Response to reviewers.docx [file pone.0236339.s004.docx]

**Reply to reviewers**

We thank the reviewers for the time that they have taken to review our manuscript and the helpful comments that they have made to improve the work. Please find below our point-by-point responses to their comments. For clarity, the reviewers’ comments are presented in blue normal text, while our responses are in black normal text. Any modified - or highlighted - text is presented as black and italicized.

Reviewer #1: Thank you for the opportunity to review this important piece of work. The single centre retrospective study presents original research, not elsewhere reported, comparing the ability of several mortality predicting scoring tools to predict mortality in ICU and at 90 days in Aboriginal and Torres Strait Islander peoples and non-Indigenous Australians, with sepsis. The authors report a similar predictive ability of the ANZROD, APACHE II, APACHE III, SAPS 2, qSOFA and SOFA scores in predicting death in Indigenous and non-Indigenous patients with sepsis over a 3-4 year period.

I have made several comments for the authors to consider in the attached PDF manuscript file and it may be easier for the authors to review this as opposed to me listing all of the notations here, but I outline major concerns below, in addition to minor concerns (highlighted with comments in the attached PDF).

1. Taking into account the NHMRC guideline on ethical conduct in research with Aboriginal and Torres Strait Islander peoples and communities I am curious as to whether the authors are familiar with this document and/or whether any Aboriginal and/or Torres Strait Islander peoples or communities were involved in any aspect of the study or in drafting of the manuscript?

The six core guiding principles of this guideline are key to any research undertaken with Aboriginal and Torres Strait Islander people and communities, however it was not clear from the manuscript whether the authors have considered these principles:
• respects the shared values of Aboriginal and Torres Strait Islander Peoples
• is relevant for Aboriginal and Torres Strait Islander priorities, needs and aspirations
• develops long-term ethical relationships among researchers, institutions and sponsors
• develops best practice ethical standards of research.

I recommend the authors consider these guidelines and incorporate a reflection of how they have considered these guidelines into the manuscript; methods, results and discussion. If there has not been any consultation with Indigenous persons regarding the research, this is a major limitation (and justification for rejection of the manuscript) and should be added to the limitations within the discussion. I suggest, if the journal has not already done so, that they consider enlisting an Aboriginal and/or Torres Strait Islander researcher to review this article and provide comment from an Aboriginal and/or Torres Strait Islander perspective. I am happy to make recommendations of appropriate reviewers, if needed.

Response: We agree with the reviewer that it is essential that research which examines Aboriginal and Torres Strait Islander issues is respectful, responsible, and of benefit to Aboriginal and Torres Strait Islander people and communities. We are familiar with the document that the reviewer highlights and hope that the work we have produced reflects the principles outlined in that document.

It is important to note that one of the authors – Associate Professor Venessa Curnow - is an Ait Keodal and Sumu (Torres Strait Islander) woman and the Executive Director of Aboriginal and Torres Strait Islander Health at Torres and Cape Hospital and Health Service. She is also a board member of the Congress of Aboriginal and Torres Strait Islander Nurses and Midwives and former board member of the National Congress of Australia's First Peoples.

Associate Professor Curnow had a critical role in the writing of this manuscript. She feels that the manuscript highlights a clinical issue that is of importance to Aboriginal and Torres Strait Islander Australians and - in addition - provided cultural advice about the way this information was presented.

Associate Professor Curnow is the professional lead for all Aboriginal and Torres Strait Islander Health Practitioners and Health Workers within the Torres and Cape Hospital and Health Service has been a strong advocate for the building of local Aboriginal and Torres Strait Islander research capacity. She has also ensured that previous work performed at Cairns Hospital which examines Aboriginal and Torres Strait Islander health issues is disseminated appropriately through local networks to actually have an impact on models of care. She emphasised that Aboriginal and Torres Strait Islander Australians recognise that they suffer a disproportionate burden of sepsis and are keen to identify solutions that might reduce this impact.

Consumer and Community Engagement is within her current portfolio which includes experienced based co-design with Aboriginal and Torres Strait Islander Peoples from across 60 different Traditional Owner groups in the Torres and Cape Hospital and Health Service catchment. Through this work she also develops and strengthens partnerships with Aboriginal and Torres Strait Islander Community Controlled Health Organisations and other Aboriginal and Torres Strait Islander organisations within the region.

2. The authors state that the primary objective of the study was to compare mortality predicting scoring systems in Indigenous and non-indigenous Australians. However, I feel the methods and results also emphasize understanding important characteristics of the populations and comparisons of these populations and that this should be considered.
I would be happy to review an edited version of the manuscript should the journal be able to seek peer review from an Aboriginal and/or Torres Strait Islander researcher.

Response: We agree with the reviewer that the dataset contains demographic, epidemiological, clinical and laboratory data all of which have a story to tell. Indeed, this is the basis of several other reports that we are preparing. However, if we try and present all this information in a single manuscript, we are concerned that the individual messages may be lost.

This study was designed to focus only on the utility of prediction scores. We felt it was important to present the cohort’s demographic and clinical data so that the reader could draw some conclusions about the generalisability of our findings. While further discussion about the characteristics and comparisons of the populations is warranted, they will be presented in future projects that examine those themes specifically.

Comments contained within the pdf

Line 78: Suggest the authors update the introduction to reflect publication of the Lancet Sepsis GBD study which provides more recent estimates of disease burden.

Response: We thank the reviewer for highlighting this updated reference. We have revised the paper accordingly (lines 102-103).

Line 114: Not previously abbreviated, please spell out.

Response: We thank the reviewer for highlighting this oversight. We have revised the paper accordingly (lines 118-119).

Line 114: Provide the names for these codes, at least in a supplement or table.

We thank the reviewer for highlighting this oversight. We have revised the paper accordingly (lines 119-120).

Line 116: such as and were they pre-existing?

Response: We thank the reviewer for highlighting this oversight. We have revised the paper accordingly (lines 123-125).

Line 118: Needs a reference if this is what is defined as hazardous.

Response: We have added a reference as requested, although technically it is not required. We are simply presenting the cut-off that we used in this dataset (line 126).

Line 125: APACHE = acute physiological and chronic health evaluation is the terminology I am familiar with.

Response: In the original publication in Critical Care Medicine in 1985 which we reference, Knaus *et al* - the authors of the APACHE-II score - report that the acronym APACHE stands for Acute Physiology And Chronic Health Evaluation.

Line 127: Acronym should be spelled out first use.

Response: We thank the reviewer for highlighting this oversight. We have revised the paper accordingly (lines 134-135).

Line 131: how was death at 90 days determined?

Response: We thank the reviewer for highlighting this oversight. We have amended the paper to make this clearer (lines 138-139).

Line 140: This paragraph is quite unclear and this is reflected in the results presented in the tables. The analysis needs to be further explained

and

Line 141: Description of Liu's method would be helpful.

Response: Area under the receiver operating characteristic curve is a very standardised way of determining the ability of a test or score to discriminate between two patient states (alive or dead, diseased or non-diseased). Indeed, reviewer three appears comfortable discussing the AUROC results. Similarly, Liu’s method is a common way to define the optimal cut-point of a score/test to maximize its sensitivity and specificity. Detailed discussions of these statistical techniques are beyond the scope of the article, but a reference has been attached for both for the interested reader (references 21 and 22).

Line 142: . missing

Response: We thank the reviewer for highlighting this oversight! We have revised the paper accordingly (line 150).

Line 153: but quite different % of M:F when looking at indigenous status.

Response: It is true to note that 57% of the Indigenous patients were male and this is something that we may examine in some of the other planned manuscripts using this dataset. Indeed, we recognise that Aboriginal and Torres Strait Islander men experience significantly worse health outcomes and are the most marginalized and disadvantaged population group in Australia. We have expanded our discussion about prevention of infection through improvements in primary care (lines 341-349).

Lines 160: pre-existing. What defines a significant comorbidity - this was not stated in methods.

Response: We thank the reviewer for highlighting this oversight. We have revised the methods accordingly (lines 123-125). Hopefully, this addresses the reviewer’s concerns.

Lines 166: ? excluded as per methods.

Response: We thank the reviewer for clarifying this point. The only episodes of sepsis that were excluded were recurrent episodes that occurred in patients **during the study period** (1 January 2014 to 30 June 2017). If a patient had several episodes within the study period, only the first episode was included (to reduce the potential bias that this might lead to). If patients had a previous episode of sepsis - prior to the study period - they were able to be enrolled. We have revised the manuscript to highlight this (line 178).

Line 174: ? Clinical characteristics of sepsis

Response: We agree with the reviewer “Nature of the sepsis” was a clunky title. We have revised the paper accordingly (line 194).

Line 175: should report numbers here for the groups.

Response: The numbers are presented in table 2. We have not presented them here again in the text as we feel that it would be duplicative, although we would be happy to if the Editor felt that this improved the legibility of the paragraph.

We may have contributed to this problem by not including the table immediately after the paragraph as advised in the instructions to authors. This would have allowed the reviewer to inspect the relevant data more easily.

Lines 179: Is this overall or just among Indigenous patients. Needs to be stated more clearly.

Response: We thank the reviewer for highlighting this oversight. We have revised the paper accordingly (line 199).

Line 179: most commonly isolated

Response: This is really a question of style, but we have revised the text as suggested (line 199).

Line 183: ? add numbers here.

Response: Similar to the comment above, we have not presented the numbers in the text as well as in table 3 as this would be duplicative. We would be happy to do so if the Editor felt that this improved the legibility of the paragraph.

Line 186: superlative - delete.

Response: We thank the reviewer for highlighting this. We have revised the paper accordingly (line 218).

Line 187: Reword this sentence to.... There was no significant difference in requirement for renal replacement therapy.

Response: We understand what the reviewer is saying here – the p value is greater than 0.05 so the difference is not statistically significant. However, this is likely to be a type 2 error in this small sample.

Indigenous patients were more likely (statistically) to have a history of renal dysfunction and biochemical evidence of renal dysfunction. If the sample were larger, we would expect that Indigenous patients would be more likely to require RRT. Therefore, we believe that highlighting the raw numbers is worthwhile.

However, to address the reviewer’s comment, we now add that this is a small sample which hopefully relays the fact that the lack of statistical significance is because the study is underpowered (line 220). This is a point that we also make in the expanded limitations discussion (lines 334-335).

Line 196: who

Response: We thank the reviewer for highlighting this oversight. We have revised the paper accordingly (line 236).

Line 198 with sepsis

Response: We thank the reviewer for highlighting this source of potential confusion. We have revised the paper accordingly (line 238). We have also made a correction to the number of patients that were local residents (416 not 442).

Line 203 Unclear how death at 90-days was determined. Please add to methods.

Response: We thank the reviewer for highlighting this oversight. We have revised the methods to address this issue (lines 138-139).

Line 246: More likely to be women too!

Response: We agree with the reviewer there are many aspects the healthcare of Aboriginal and Torres Strait Islander Australians that could be examined more closely. In this paper we are focussing on the disease severity scores, although we have expanded the discussion to discuss ways that we might improve access to primary health for both Aboriginal and Torres Strait Islander men and women (lines 346-349).

Line 268: it's retrospective observational, not a trial.

Response: We thank the reviewer for highlighting this oversight. We have revised the paper accordingly (line 329).

Line 271: , with respect to......

Response: This information is presented in lines 95-98 and 307-313, so we did not repeat it again.

*“While there are relatively few published data examining the ICU care of Australian Indigenous patients with sepsis specifically, there are several studies that have examined the clinical characteristics and outcomes of Indigenous patients admitted to ICU (12-15). These studies are strikingly similar and show that Indigenous patients with critical illness are younger, have greater comorbidity and are more frequently admitted from remote locations than non-Indigenous patients. These studies also universally show that there is no difference in the proportion of Indigenous and non-Indigenous patients who die in the ICU.”*

However, it is evident that this it was not sufficiently clear, so we have now added *“as previously noted”* to refer the reader back to the prior text (line 332).

Lines 274: What about cultural and political health system influencing factors that determine health in Aboriginal and Torres Strait Islander peoples. In Primary Care, Community Controlled Health Organisations have been effective as they enable self-determination. Is there a place to also highlight the importance of culturally safe spaces for First Nations people to seek health. What about the definition of health to Aboriginal and Torres Strait Islander peoples, which extends well beyond physiological health parameters. I think these are important points to make in the discussion.

Response: We thank the reviewer for highlighting these important issues. The challenge of improving Aboriginal and Torres Strait Islander health in Australia is obviously complex and multifaceted. There are many medical, sociocultural, and political issues that might be discussed. However, our focus in this paper was on the relatively specific and practical issue of the utility of prediction scores in Indigenous Australians admitted to ICU with sepsis.

However, we recognise that the development of sepsis is, in many cases, due to a failure in primary care. While we do not want to distract from the main point of the paper, we have expanded the discussion to acknowledge the role of primary health in improving Aboriginal and Torres Strait Islander peoples’ access to care. We also now highlight the potential impact of Community Controlled Health Organisations, which we agree have an important role to play (lines 341-349).

Reviewer #2: Although Aboriginal and Torres Strait Islander is longer to write it is the preferred terminology for Aboriginal and Torres Strait Islander people in Australia. Please change throughout the paper. Another preferred terminology that you might like to use is Australia's First Nation people.

Response: We agree with the reviewer that Aboriginal and Torres Strait Islander people is the preferred terminology. However, Indigenous Australian is also a frequently used term and one that very few Aboriginal and Torres Strait Islander people actually find inappropriate, including our co-author A/Prof Venessa Curnow, an Ait Keodal and Sumu (Torres Strait Islander) woman. Furthermore, we use the word “Indigenous” 131 times in the manuscript. If we were to replace this with Aboriginal and Torres Strait Islander people or Australia’s First Nation people, it would impact significantly on the length and legibility of the article.

However, to acknowledge this point and address the reviewer’s concerns, we have amended the text to read Aboriginal and Torres Strait Islander people (hereafter respectfully referred to as Indigenous Australians) (lines 93-94)

Line 186 page 8
Please indicate what supportive care means
Response: We acknowledge that is a loose term. We have refined it to Intensive care support (line 217).

Line 221 page 10
Different font colour
Response: We thank the reviewer for highlighting this oversight. We have revised the paper accordingly.

Line 231 page 10
“It is the only part of Australia which has the homelands of both Aboriginal and Torres Strait Islander Australians, peoples that are frequently conflated but who are ethnologically quite distinct”

Has “conflated” been recognised and confirmed by the community members? This can’t be just assumed. If this has not had input from community members, please remove word from this sentence.
“ethnologically” This word is a derogative term please remove it.
This sentence needs rewording to capture Aboriginal and Torres Strait Islander people’s distinct cultural groups.

Response: We thank the reviewer for raising this important point. In fact, this sentence (lines 290-292) was composed by A/Prof Curnow an Ait Keodal and Sumu (Torres Strait Islander) woman. She is also the professional lead for all Aboriginal and Torres Strait Islander Health Practitioners and Health Workers within the Torres and Cape Hospital and Health Service.

Ethnology is a scientific term describing the branch of anthropology that compares and analyses the characteristics of different peoples and the relationships between them. So, it seems to us – and it was a Torres Strait Islander woman who composed this sentence - that ethnologically is a reasonable choice of words and not derogative.

We would be happy for the Editor to suggest another word – socioculturally? anthropologically? – if they feel it appropriate.

Reviewer #3: The information provided with this article is not new to anyone familiar with the health determinants of the Australian Indigenous population in North Australia.
However, I think it is important to publish this research as it will stress/reiterate the need for more research and also the feasibility of using scoring systems for comparison of patient for multicentre research as well as country wide and internationally.

Response: We thank the reviewer for their positive comments.

Abstract:

Materials and methods: Cairns is referred to as a “tertiary-referral Hospital”. According to QH documentation and the Service’s homepage Cairns Hospital is not a referral hospital (the homepage states: “main referring hospitals: The Townsville Hospital 344 km, Royal Brisbane and Women’s Hospital 1680 km). So I suggest to use the term Base Hospital or a similar term, or to refer to the Clinical Services Capability Framework of Queensland Health 9CSCF)

Response: We respectfully disagree with the reviewer’s assertion that Cairns Hospital is not a tertiary referral hospital.

A tertiary referral hospital is a major hospital that provides an array of specialist services including obstetrics and gynaecology, paediatrics, medicine (including advanced cardiology services) and surgery. It also has both adult and neonatal intensive care services. In Queensland Health’s most recent clinical services plan, it is referred to as a tertiary referral hospital.

<https://www.health.qld.gov.au/__data/assets/pdf_file/0036/695358/csp-web-spreads.pdf>

Cairns Hospital was previously known as Cairns Base Hospital, but it has not been known as Cairns Base Hospital since 2013.

It does refer some patients to other hospitals - particularly those requiring transplantation, neurosurgery or cardiothoracic surgical services - but this does not mean it is not a tertiary referral hospital.

Results: figures as expected

Response: We take this to mean that the reviewer was not surprised by the findings.

Conclusions: “supporting their use”

I think this needs further clarification! Whilst not discussed in the paper, someone just reading the abstract may think the scores can be used to tailor treatment / intervention for the individual patient. To my opinion none of the scores showed an AUROC sufficient to be used as a prognostic tool for an individual for death, so to decide about how far to escalate the therapeutic effort for the individual patient and when to stop escalating therapy (for example, whom to ventilate, where to use ECMO, CHHV, increase inotropes). The closest score is APACHE-III with an AUROC of 0.84. This also is not surprising and would not need a score – it probably is just common sense that the more interventions you need to keep a patient alive, the more likely they are to die.

Of course, for the purpose of comparing patients for benchmarks, activity comparison and funding, studies, the scoring systems definitely are useful, as stated in the discussion P 12 266-267.

I would suggest to change the sentence in the abstract to “… supporting their use to compare the efficacy of interventions …”

Response: The reviewer is absolutely correct to note that these scores have almost no utility in the individual management of patients. Indeed, this is why many of them were calculated retrospectively for the purposes of this study.

However, they do have utility in the benchmarking of ICU performance and in clinical trials to ensure that patients in different arms of an intervention study.

We make this point in the introduction (lines 87-92) and discussion (lines 326-328).

P4-5 Introduction

There is no definition of sepsis provided.

Response: Sepsis is not defined in so many words, but this is true of many of the concepts discussed in the manuscript (Intensive Care Unit, immunocompromised, remote location, drug resistant organism *etcetera*). Some of this is assumed knowledge for the reader who, we imagine, will have some interest in the subject.

However, in the revised methods section, we do describe quite precisely how we defined sepsis for the purposes of this study (lines 118-120).

P8 Clinical and laboratory findings

Metabolic acidosis (Bicarbonate 0.001, BE 0.004) must be more common if renal impairment is more common Crea/ eGFR P << 0.001), so Metabolic Acidosis is not an independent variable, may be worth to comment.

Also, probably worth to comment: no significant abnormalities in LFTs, Platelet count (there is a typo in the table on P 18 that needs correction), Albumin for both the Indigenous and non-Indigenous patient groups.

Response: The reviewer is almost certainly correct to link the metabolic acidosis to renal dysfunction, one of the most common causes of acidosis in the hospital setting. However detailed discussion of the individual laboratory variables is probably beyond the scope of the manuscript. The informed reader – like the reviewer – will quickly see these patterns and draw the same conclusions.
