# Supplementary material for: The applicability of commonly used predictive scoring systems in Indigenous Australians with sepsis: An observational study
Source: PLoS One. 2020 Jul 22;15(7):e0236339. doi: 10.1371/journal.pone.0236339 (PMC7375531; doi:10.1371/journal.pone.0236339)
Supplement: Attachment — Submitted filename: Response to Reviewers.docx [file pone.0236339.s005.docx]

Reviewers' comments:

Reviewer's Responses to Questions

**Comments to the Author**

1. If the authors have adequately addressed your comments raised in a previous round of review and you feel that this manuscript is now acceptable for publication, you may indicate that here to bypass the “Comments to the Author” section, enter your conflict of interest statement in the “Confidential to Editor” section, and submit your "Accept" recommendation.

Reviewer #2: (No Response)

Reviewer #3: (No Response)

2. Is the manuscript technically sound, and do the data support the conclusions?

The manuscript must describe a technically sound piece of scientific research with data that supports the conclusions. Experiments must have been conducted rigorously, with appropriate controls, replication, and sample sizes. The conclusions must be drawn appropriately based on the data presented.

Reviewer #2: Yes

Reviewer #3: Yes

Response: We are happy that the reviewers believe that the manuscript is technically sound.

3. Has the statistical analysis been performed appropriately and rigorously?

Reviewer #2: Yes

Reviewer #3: I Don't Know

Response: We are happy that the reviewers have not raised any concerns about the statistical analysis.

4. Have the authors made all data underlying the findings in their manuscript fully available?

The [PLOS Data policy](http://www.plosone.org/static/policies.action#sharing) requires authors to make all data underlying the findings described in their manuscript fully available without restriction, with rare exception (please refer to the Data Availability Statement in the manuscript PDF file). The data should be provided as part of the manuscript or its supporting information, or deposited to a public repository. For example, in addition to summary statistics, the data points behind means, medians and variance measures should be available. If there are restrictions on publicly sharing data—e.g. participant privacy or use of data from a third party—those must be specified.

Reviewer #2: Yes

Reviewer #3: Yes

Response: We are happy that the reviewers have had the opportunity to review all the data supporting our study.

5. Is the manuscript presented in an intelligible fashion and written in standard English?

PLOS ONE does not copyedit accepted manuscripts, so the language in submitted articles must be clear, correct, and unambiguous. Any typographical or grammatical errors should be corrected at revision, so please note any specific errors here.

Reviewer #2: Yes

Reviewer #3: Yes

Response: We are happy that the reviewers have found our manuscript intelligible.

6. Review Comments to the Author

Please use the space provided to explain your answers to the questions above. You may also include additional comments for the author, including concerns about dual publication, research ethics, or publication ethics. (Please upload your review as an attachment if it exceeds 20,000 characters)

Reviewer #2: This is a very important subject for Aboriginal and Torres Strait Islander people and this manuscript has covered the subject well. The manuscript is well written and should be published.

Response: We are happy that reviewer #2 has recommended publication of our manuscript.

Reviewer #3: I think there is a typo in line 278.
shouldn't it read: "... and have a significantLY HIGHER comorbidity ...

Response: We agree with the reviewer that the sentence was poorly constructed and contained an additional typographic error. We have amended it in the revised manuscript (lines 277-278).

Two other comments with the second read of the revised subscription (not necessarily for correction but if a revision is requested to consider:

1) The title of the manuscript focusses on predictive scoring systems.
The conclusion however discusses in its first sentence the case fatality and that the Indigenous patients die at a younger age. Whilst this is a very important fact / statement (and not new), this was not the question of this study. The conclusion in regard to the question of the study is mentioned (like a dependend variable) in the second sentence / statement. Would it be worth to swab them?

Response: We understand the point that the reviewer is making here. The focus of the study was severity scores, so the reviewer is suggesting that severity scores be mentioned in the first line of the conclusion.

This is largely a question of style, however we would also argue that

1. As death is the dependent variable for prediction scores, it makes sense to describe the variable in more detail, to address the possibility of confounding factors.

2. As the reviewer notes this is an important fact and while sadly not a new observation for Australian readers, it will help the International reader - who may not be familiar with this information – in their interpretation of our findings.

2) I still think that from 2014 - 2017 Cairns Hospital was NOT addressed as a Tertiary Hospital but as a retrieval "base" hospital. This is reflected by the CSCF (and also the ACEM accreditation for their Emergency Department) I know that there is a recent push / move towards addressing Cairns Hospital as a Tertiary facility (despite the lack of especially neurosurgery and cardiothoracic surgery)- but this was definitely not applicable at the time where the study data were collected

Response: We understand the point that the reviewer is making here. However we would argue that a hospital with advanced diagnostics (MRI, PET scanning, molecular laboratory) and which has over 500 beds (including adult and neonatal ICU) and which provides specialist medical, surgical, psychiatric, obstetric, paediatric and cancer care (including radiotherapy) is a tertiary referral hospital.

It would appear to satisfy most definitions of the term.

<https://en.wikipedia.org/wiki/Tertiary_referral_hospital>

It is true that there is no neurosurgery or cardiothoracic surgery but many of the authors have trained in metropolitan tertiary centres in Australia which have no paediatric, obstetric or neonatal facilities and yet they would still be recognised as tertiary centres.

We think that most practicing clinicians would understand what a tertiary centre is and would agree with our assessment. We feel that this is more than a semantic argument; it is is important that the reader can understand the context of the patient’s care and the impact that this might have had on the mix of patients in the ICU and their case-fatality rates (the dependent variable for the severity scores).

We will leave it to the Editor to adjudicate. If the Editor were unpersuaded by our arguments, we would be happy for the abstract which currently reads as

The study was performed at an Australian tertiary-referral hospital between January 2014 and June 2017, and enrolled consecutive Indigenous and non-Indigenous adults admitted to ICU with sepsis.

To be replaced with

The study was performed at Cairns Hospital, in tropical Australia, between January 2014 and June 2017, and enrolled consecutive Indigenous and non-Indigenous adults admitted to ICU with sepsis.
